# Comparison of flexion relaxation phenomenon between female yogis and matched non-athlete group

**DOI:** 10.1186/s13102-022-00406-4

**Published:** 2022-01-22

**Authors:** Marzyeh Ramezani, Amin Kordi Yoosefinejad, Alireza Motealleh, Mohsen Ghofrani-Jahromi

**Affiliations:** 1 Physical Therapy Department, School of Rehabilitation Sciences, Shiraz University of Medical Sciences, Chamran Blvd., Sheikh Abivardi 1, Shiraz, Iran; 2 Rehabilitation Sciences Research Center, Shiraz University of Medical Sciences, Shiraz, Iran; 3 Department of Medical Physics and Biomedical Engineering, Shiraz University of Medical Sciences, Shiraz, Iran

**Keywords:** Yoga, Flexion relaxation phenomenon, Electromyography, Kinovea

## Abstract

**Background:**

Trunk flexion is a common exercise during daily activities. Flexion relaxation phenomenon (FRP) occurs during forward bending in which there is a sudden silence of erector spinae (ES) muscles. The pattern of forward bending differs in yoga practitioners. This learned pattern probably predisposes yogis to injuries. The hypothesis of this study was that FRP differs in yogis in comparison to non-yogis individuals.

**Methods:**

This observational cross-sectional study was performed on 60 women assigned into yogis and non-athlete groups. Each participant was asked to bend forward and then return to the initial position. ES activity was recorded at L3 level, 4 cm from mid line during the trial. Trunk inclination and lumbar flexion angles were calculated at FRP onset and cessation moments.

**Results:**

The FRP occurred in 80% of yoga practitioners in comparison to 96.7% in the control group. Trunk inclination angle was significantly greater at FRP initiation in yogis compared to control group. Lumbar flexion angle was not different between the groups.

**Conclusions:**

It is concluded that the altered pattern of forward bending observed in yogis may change patterns of ES muscles activity if it becomes part of a person's daily lifestyle which might predispose these muscles to fatigue and subsequent injuries; however, further studies are warranted for clarification.

## Background

Flexion relaxation phenomenon (FRP) is defined as reduced or sudden silence in myoelectrical activity of erector spinae (ES) muscles during trunk forward bending that occurs in healthy individuals [1]. During forward bending, ES muscles control and coordinate the movement; nevertheless, once tension increases in non-contractile tissues, they take over the control of trunk forward bending and the activity of ES muscles is not required [1]. Non-contractile tissues and their receptors have an important role in activating or deactivating ES muscles during forward bending. Articular and ligament receptors provide information about equilibrium between gravity and tensile force in non-contractile tissues to brain and the brain inhibits muscle activation [2, 3]. Activity or inactivity of ES muscles depends upon many factors including forces imposed on trunk, position of pelvis, speed of trunk, loading rate, repletion of activity and fatigue of these muscles [1, 2, 4]. During the occurrence of FRP, the role of ES muscles is to shift the torque generated by the trunk on the lumbar region to the passive adjacent tissues. Thus, FRP might help preservation of energy by preventing ES muscles from sustained prolonged contraction [1]. Previous studies on FRP demonstrated that muscle activity reduced by 78% in full flexion [5].

ES muscles are unloaded following the enhanced activity of non-contractile tissues to control forward movement of trunk [6]. It is known that excessive and prolonged activity of ES muscles induces fatigue and decreases the ability of non-contractile tissues to stretch that would results in lumbar dysfunction. Comparing individuals with low back pain (LBP) to healthy ones during forward bending revealed an increased) activity of ES muscles in people with LBP indicating the compensation for the decreased ability to resist the tension of stretched passive tissues [7, 8]

Yoga is a popular sport originated from India about more than 5000 years ago [9]. It comprises physical, mental, emotional and psychological aspects of life [10]. Yoga was claimed to increase strength, endurance, flexibility, balance and motor coordination [11, 12]. Studies conducted on common yoga-related injuries included supra-spinatus tendon rupture, glenoid labrum tears and LBP [9]. Generally, 62% of yoga practitioners have had at least one episode of musculoskeletal problems [13]. Previous studies investigated the biomechanical demands posed on the lower extremities of yoga practitioners [14, 15]; however, no study yet, has evaluated the FRP occurrence in yogis [16]. In yoga, there is a set of positions called Asana involving forward bending. The yoga practitioner is instructed to stand with feet slightly apart and bend forward while keeping knees straight. The practitioner is asked to intentionally preserve lumbar lordosis and tilt pelvis anteriorly while bending forward and to start the motion from hip and then bend forward as much as he/she can [17]. Yogis may habituate with this pattern of bending during activities of daily living. On the other hand, biomechanical studies indicated that the normal rhythm of motion during trunk flexion is from proximal to distal which is known as lumbopelvic rhythm and the absence of this rhythm may lead to muscle fatigue and resultant LBP [2]. Plausibly, we hypothesized that the FRP in yogis might be different from those of healthy subjects. Thus, the objective of the present study was to evaluate FRP in yoga practitioners and to compare it with those of non-yogis.

## Methods

### Subjects

This observational cross-sectional study was conducted between July 2018 and March 2019 at motion lab of research center of school of rehabilitation sciences (Shiraz University of Medical Sciences, Shiraz, Iran). Ethical approval for the study was granted by the Ethics Committee of Shiraz University of Medical Sciences, in accordance with the declaration of Helsinki and subjects provided written informed consent (Ethics number: IR.SUMS.REC. 1395.S159). The minimum sample size was calculated as 28 subjects in each group (healthy group, yogis group) based on a previous study [2], regarding offset time of lumbar ES muscles as the primary outcome measure (d = 6 ms), considering an alpha level of 0.05 and a beta level of 0.2. Yoga practitioners were recruited through yoga clubs of Shiraz (south of Iran), using convenience sampling. Female yogis fulfilling the following criteria were considered eligible to participate in the study: aged between 18 and 40 years and practicing yoga for at least 6 months under supervision. The exclusion criteria were history of pain, injury or surgery of back, pelvis, and lower extremities during the previous 6 months, cardiorespiratory, neuromuscular or musculoskeletal disorders. Low back pain while practicing yoga, tight hamstrings and individuals in menstrual phase at the test day were also excluded. Control group was matched for age and body mass index (BMI). The control group was recruited through advertisement or direct interview from community-dwelling women.

### Instrumentation

The activity of ES muscles was recorded using ME 6000 electromyography telemetry system, 16-channel (Mega Electronics Ltd., Kuopio, Finland). EMG data were recorded with a sampling frequency of 1000 Hz, band pass filtered between 8 and 500 Hz, 1 µV noise referred to input, and common mode rejection ratio of 110 dB. The input impedance was 10^9^ KΩ. Data were transmitted via a 14-bit analogue-to-digital convertor and stored for later processing. EMG recording was based on SENIAM guidelines. Recording was performed on dominant (right) side at L3 level; 4 cm from mid line using pre-gelled Ag-AgCl disposable self-adhesive disc electrodes with a diameter of 20 mm aligned longitudinal to muscle fibers (Medico lead-lok brand, India). The interelectrode distance was set 2.5 cm. The electrode placement was performed in forward trunk flexion in order to prevent electrode slippage.

Angular variables, including trunk inclination angle and lumbar flexion angle, were estimated by a digital camera (Sony, xr550 two-dimensional imaging camera) with a sampling rate of 25 frames per second. The camera was placed 3 meters from the participant at waist level with direct view of participant’s right side in sagittal plane. To measure inter-segmental angles, retro-reflective calibration markers with a diameter of 19 mm were attached to right side greater trochanter, lower ribs on mid axillary line and the middle point of iliac crest.

EMG data and video were synchronized by a triggering device. It consisted of a light emitting diode tuning on and off simultaneously with recording of EMG signals.

### Protocol

Participants were instructed not to practice yoga for 24 h before test. The skin was shaved, cleaned, and lightly abraded with alcohol prep pads before electrode attachment. To record the muscle activity of lumbar ES, participants were asked to wear a shirt that was open posteriorly. The wires were fixed with anti-allergic adhesive tape to prevent motion artefact or cable shaking-induced noise.

Before commencing the trial, participants were required to practice the desired task for several times to achieve stable fluidity and rate of motion. Participants were instructed to perform the flexion phase within 2 s, then remain in full flexion for 2 s and next return back to initial position within 2 s. Each participant performed 3 trials with one-minute rest among the trials. A metronome was used to regulate the speed of movement. To obtain a standard position of head and neck at the beginning of trials, a cross sign proportional to individual’s height (level with eyes) was mounted on the opposite wall and the individual gazed at the sign.

### Analysis

**Fig. 1:**
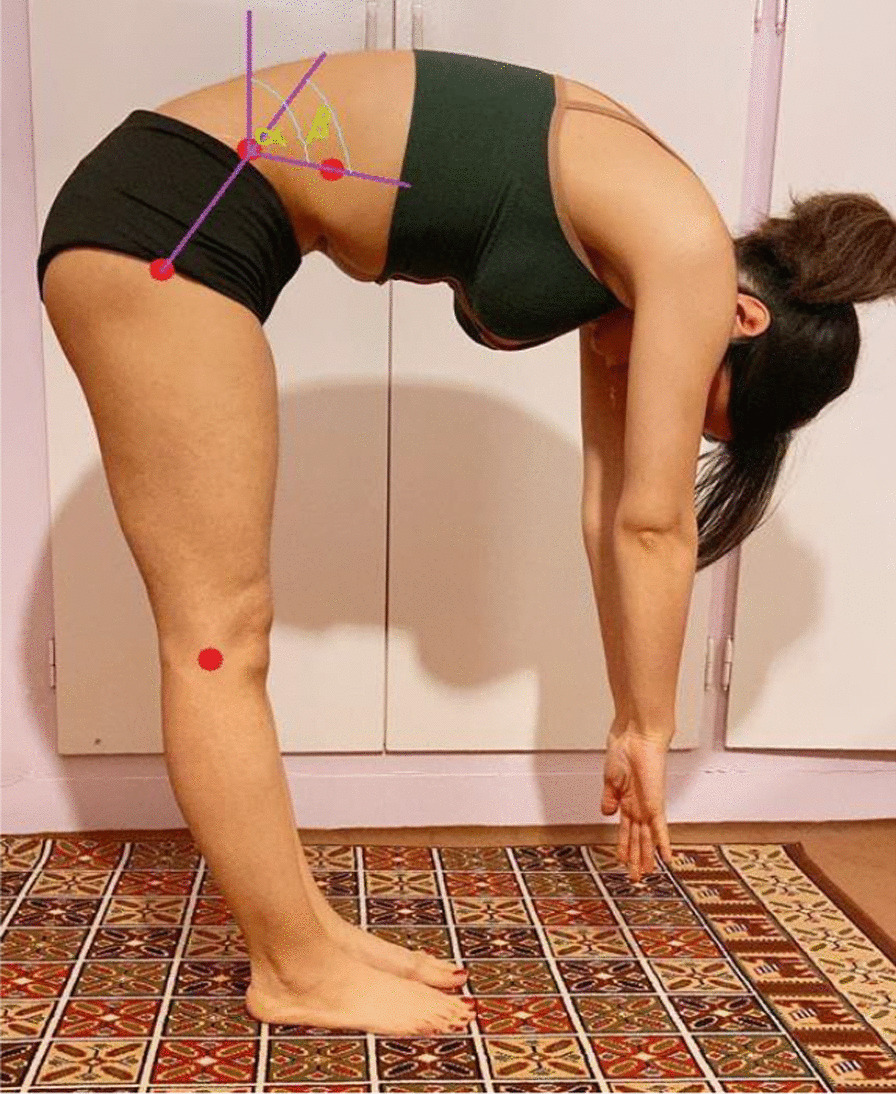
Determination of trunk inclination and lumbar flexion angles

Trunk inclination angle and lumbar flexion angle were recorded according to Solomonow [18]. Trunk inclination angle is defined between the line connecting iliac crest marker to lower ribs marker and perpendicular line through the marker on iliac crest. Lumbar flexion angle is defined as the angle between the line connecting greater trochanter to iliac crest marker and the line connecting lower rib marker to iliac crest. Kinovea software (Experimental version, 0.8.27) was used to determine the concerned frames and to measure angles (Figure 1). The reliability of Kinovea software was previously confirmed for measuring angles [19].

Raw EMG data were saved as ASCII files and imported from MEGAWIN software version 3.0 to MATLAB software (r2015b, manufactured by Met Works America Co.) for analysis.

**Fig. 2:**
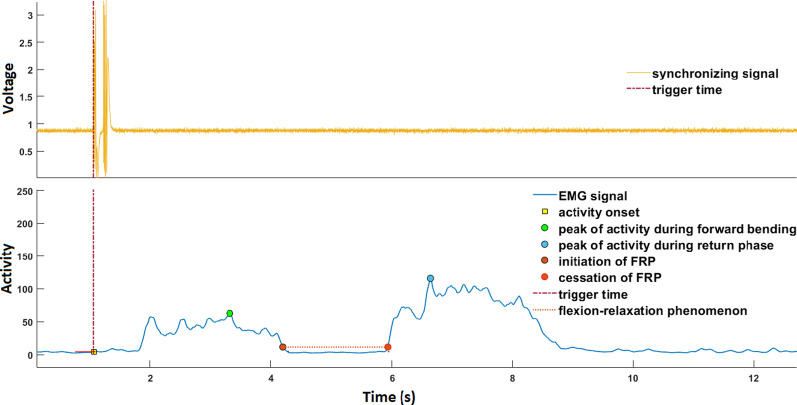
Peaks in the activity of erector spinae muscles in bending and return phases

Raw EMG signals were evaluated visually and then, were fully rectified. Data were filtered using a band pass frequency between 20 and 500 Hz. Onset time was defined when muscle activity continued for at least 25 ms and the amplitude was at least three times of standard deviation from baseline [20]. EMG signals of right ES muscles showed two peaks of activity; one for flexion phase and the other for return phase (Fig. 2).

Peak muscle activity was calculated during bending and returning back phases by root mean square formula with a time constant of 100 ms. The moment that the muscle activity was less than 10% of peak activity during bending phase was regarded as the initiation of FRP and the moment it reached to more than 10% of peak activity during return phase was regarded as cessation of FRP. Initiation a cessation of FRP was calculated between two peaks of the activity of ES [21, 22].

The other outcome measures were trunk inclination and lumbar flexion angle at the moment of initiation and cessation of FRP.

### Statistical analysis methods

Data were analyzed with SPSS version 16.0 (SPSS, Chicago, IL, USA). Normal distribution of data was confirmed with Kolmogorov-Smirnov test. Chi-square test was used to determine the incidence of FRP. Independent sample t-test was used to compare data between the groups. The acceptable value was obtained at the level of *P* < 0.05.

## Results

Sixty women participated in the study. Thirty were yogis (age: 31.56 ± 5.90 years, BMI: 24.39 ± 3.01 kg/m^2^) and 30 were healthy matched women (age: 30.56 ± 5.59 years, BMI: 23.76 ± 2.64 kg/m^2^) as control group. No significant difference was observed in demographic data between the groups ]age: t(58) = 0.54, *P* = 0.59, 95% CI (− 2.17, 3.77), BMI: t(58) = 0.85, *P* = 0.39, 95% CI (− 0.84, 2.09)].

There was a significant difference (*P* = 0.04) between yogis (24/30, 80%) and control group (29/30, 96.7%) for the occurrence of FRP. It is worth noting that FRP did not occurred in 6 yogis and one subject in control group.

Trunk inclination angle was significantly greater at FRP initiation moment in yogis compared to control group during forward bending. Lumbar flexion angle was not significantly different between the groups during forward bending at the initiation moment of FRP. During return phase, trunk inclination angle was significantly greater in yogis in comparison to control group; however, lumbar flexion angle was not significantly different between the groups (Table 1).

**Table 1:** Comparing trunk inclination and lumbar flexion angles between yogis and control group at the time of initiation and cessation of FRP

Variable	Yoga group (n = 24) mean (SD)	Control group (n = 29) mean (SD)	*P* value
*FRP initiation*			
Trunk inclination angle	104.25 (18.29)	59.03 (14.11)	< 0.001*
Lumbar flexion angle	38.41 (10.79)	36.13 (11.92)	0.47
*FRP cessation*			
Trunk inclination angle	118.62 (15.28)	87.89 (15.90)	< 0.001*
Lumbar flexion angle	44.00 (12.57)	44.17 (10.00)	0.96

*FRP* Flexion relaxation phenomenon

*Significant level at *P* < 0.05

## Discussion

Yogis maintain lumbar lordosis and then bend forward with a greater reliance on hip joint. Our results revealed a delayed onset of FRP in yogis in comparison to control group during forward bending. Also, ES muscles were activated earlier in yogis in comparison to control group during return phase. Absence of FRP occurred significantly more in yogis than control group. Lack of FRP was previously reported in some previous studies. Shirado et al. conducted a study on 25 healthy people and 20 patients with chronic low back pain to investigate the occurrence of FRP and to compare it between the groups. They showed that FRP did not occur in chronic back pain group, in contrast to healthy subjects [23]. In a study conducted by Watson et al., absence of FRP was considered as a diagnostic criterion for individuals with chronic low back pain (93% sensitivity and 75% specificity) [24]. Taken together, it can be concluded that the absence of FRP is an important index to predict injury and a diagnostic criterion for LBP. Absence of FRP was verified in six yogis (20 %) in our study. It might be attributed to special pattern of forward bending instructed to yogis. Similar to the rate of FRP absence in patients with low back pain, the yogis engage ES muscles for a longer duration which might predispose ES muscles to fatigue and predictable injuries. Future longitudinal studies are required to demonstrate if prolonged contraction of ES muscles among yogis could lead to injuries like LBP. Our findings might be attributed to some factors.

Maintaining lumbar lordosis during forward bending might be similar to an additional load bearing situation exerting extra load on ES muscles in yogis. Previously, it was shown that applying load would lead to a greater activation of lumber ES muscles and delayed FRP during forward bending [4].

Greater flexibility in yogis might be another explaining mechanism for the observed results. Shin et al. investigated the effects of flexibility on FRP and found that flexibility has a significant effect on FRP. Higher flexibility would lead to greater activity of lumbar musculature and delayed cessation of activity [25]. Chen et al. showed that higher flexibility, measured via toe-touch-test, was associated with slower occurrence of FRP [26]. It has been found that when performing identical deep trunk flexion, flexible people exhibited relatively delayed FRP compared with less flexible people [27, 28]. Chen et al. found that flexible participants have a larger range of motion for trunk flexion; when the trunk flexes forward to 90°, greater lumbar curvature is maintained, resulting in a lower degree of FRP [26].

Moreover, they found that the different FRP levels found in participants with different flexibilities can be explained by the change of lumbar curvature. The flexible participants exhibited larger lumbar lordosis from standing erect to flexing forward 90° [26]. In contrary to their findings, yogis maintain the lumbar lordosis voluntarily when performing forward bending in our study. Thus, our results could not be attributed to lumbar curvature alteration.

It is worth noting that lumbar flexion angles had no significant difference between the groups during forward bending and return phases in both initiation and cessation of FRP. Since yogis maintain lumbar lordosis during forward bending and return phases, it appears that lumber flexion had a small contribution to these movements in yogis. Maintaining lumbar lordosis might have altered normal lumbo-pelvic rhythm in yogis. We hypothesized that maintaining lumbar lordosis while bending forward could influence the cessation and onset of FRP as verified by previous literature [29].

Fatigue and overuse might be other demonstrating factors for the observed difference between the groups. Da Silva et al. compared the fatigue of back muscles in young and old people with and without chronic back pain. They concluded that fatigue occurrence was more in individuals with chronic back pain in comparison to asymptomatic persons in both young and elderly groups [30]. FRP may result in reduced back muscular energy consumption and fatigue [31]. It can be concluded that overuse of ES muscles and its subsequent fatigue are considerable injurious factors to lumbar region. It was previously confirmed that if the hyperactivity of the ES muscles was maintained for a long term, this adaptive muscle activity pattern could be problematic since as the superficial muscles stiffen the spine via sustained and augmented compression, a continuous stimulation of nociceptors in spinal structures may predispose and result in further injury [32].

Delayed onset of FRP during forward bending and earlier activity of ES muscles during return phase in yogis might be attributed to prolonged activity of ES muscles secondary to special learned pattern of bending in yogis. This might lead to overuse and subsequent fatigue in ES muscles in yogis in comparison to control group. When the lumbar curvature is close to the natural standing posture of the body, the ES bears a small load; otherwise, it bears an increasingly large load which might lead to musculoskeletal fatigue.

Moreover, it should be kept in mind that fatigue usually occurs secondary to repetitive or sustained movements. Thus, learned pattern of bending might have led to habitual overuse and subsequent fatigue following many hours of practice. However, we did not evaluate fatigue in our study and this theory requires prospective studies to compare fatigue indices among yogis and non-yogis.

Observed results might be related to other factors like creeping phenomenon and repetitive movement patterns. According to Solomonow et al., creeping phenomenon can delay the cessation of activity of lumbar ES muscles during forward bending with knees straight. Nevertheless, creep occurred following sustained lumbar full flexion for 10 minutes [18]. Since our participants maintained full flexion for only 2 s, attributing the results to immediate creep phenomenon must be interpreted with caution. However, it should be noticed if creep phenomenon might have led to permanent changes of muscle length-tension relationship in yogis. Varying amounts of creep occur through cyclic movements such as warm up and practicing in a hot yoga room [33]. Dickey et al. concluded that repetitive forward bending can affect FRP and delay the cessation of muscle activity during forward bending, but this effect was accomplished following 100 repetition of forward bending [34]. Three repetitions of forward bending in our study is unlikely to have immediate effects on FRP as both groups had the same number of repetitions. Plausibly, since forward bending is performed repetitively in yoga practice, this appears to have an impact on FRP.

Solomonow et al. declared that lumbar flexion and extension are governed by complex neuro-muscular system. The mechanism for the silence of ES muscles during trunk flexion has been proposed to result from stimulation of stretch receptors in the posterior disco-ligamentous tissues during the flexed posture, acting to reflexogenically inhibit the ES activity [1].

To the best of our knowledge, it was the first study comparing FRP between yogis and non-yogis. However, our study was not free from limitations. First, our participants were women. Thus, our results could not be generalized to male yogis. Future studies can investigate the effect of sex in FRP. Second, we did not assess fatigue indices in our study to have a direct deduction for the probable contributing mechanisms. Also, we did not evaluate creep phenomenon in our study. Future studies with the same methodology are warranted to evaluate these factors. Moreover, longitudinal studies are warranted to see whether the observed differences in FRP between yogis and non-yogis could ultimately lead to injuries among yogis and if so, to modify the pattern of forward bending and returning in yoga.

## Conclusion

Patterns of forward bending with knees straight and maintenance of lumbar lordosis alters electromyographic patterns of ES muscles activation in yogis compared to non-yogis. It might be concluded that this habitual pattern of forward bending predisposes yogis to fatigue and its subsequent complications; however, further studies are warranted for clarification.

## Data Availability

The datasets used and/or analyzed during the current study are available from the corresponding author on reasonable request.

## Abbreviations

BMI: Body mass index
EMG: Electromyography
ES: Erector spinae
LBP: Low back pain
